# Hyper-Branched Cationic Cyclodextrin Polymers for Improving Plasmid Transfection in 2D and 3D Spheroid Cells

**DOI:** 10.3390/pharmaceutics14122690

**Published:** 2022-12-01

**Authors:** Yousef Khazaei Monfared, Mohammad Mahmoudian, Claudio Cecone, Fabrizio Caldera, Sanya Haiaty, Hamid Reza Heidari, Reza Rahbarghazi, Adrián Matencio, Parvin Zakeri-Milani, Francesco Trotta

**Affiliations:** 1 Dipartimento Di Chimica and NIS, Università di Torino, Via P. Giuria 7, 10125 Torino, Italy; 2 Faculty of Pharmacy, Tabriz University of Medical Sciences, Tabriz 5166-15731, Iran; 3 Infectious and Tropical Diseases Research Center, Tabriz University of Medical Sciences, Tabriz 5166-15731, Iran; 4 Department of Pharmaceutical Biotechnology, Faculty of Pharmacy, Tabriz University of Medical Sciences, Tabriz 5166-15731, Iran; 5 Department of Applied Cell Sciences, Faculty of Advanced Medical Sciences, Tabriz University of Medical Sciences, Tabriz 5166-15731, Iran; 6 Liver and Gastrointestinal Diseases Research Centre, Faculty of Pharmacy, Tabriz University of Medical Sciences, Tabriz 5166-15731, Iran

**Keywords:** cyclodextrin hyperbranched polymers, transfection, plasmid eGFP, spheroid 3D, DAPI

## Abstract

In this article, we used monolayer two dimensional (2D) and 3D multicellular spheroid models to improve our understanding of the gene delivery process of a new modified cationic hyper-branched cyclodextrin-based polymer (Ppoly)-loaded plasmid encoding Enhanced Green Fluorescent Protein (EGFP). A comparison between the cytotoxicity effect and transfection efficiency of the plasmid DNA (pDNA)-loaded Ppoly system in 2D and 3D spheroid cells determined that the transfection efficiency and cytotoxicity of Ppoly–pDNA nanocomplexes were lower in 3D spheroids than in 2D monolayer cells. Furthermore, histopathology visualization of Ppoly–pDNA complex cellular uptake in 3D spheroids demonstrated that Ppoly penetrated into the inner layers. This study indicated that the Ppoly, as a non-viral gene delivery system in complex with pDNA, is hemocompatible, non-toxic, high in encapsulation efficiency, and has good transfection efficiency in both 2D and 3D cell cultures compared to free pDNA and lipofectamine (as the control).

## 1. Introduction

Gene therapy has emerged as a new way to change the vital cellular pathways that lead to various diseases, such as cancer and inherited disorders [1,2,3]. Viral vectors have been used for decades as the gold standard vehicle for transfecting genes with high levels of expression [4,5]. However, safety issues have remained a major concern [5,6,7] due to their ability to induce significant immunogenicity. Recently, instead of viral vectors, non-viral carriers such as inorganic nanoparticles (NPs), liposomes, and cationic polymeric NPs [8,9,10,11,12,13] have attracted the scientific community due to their capacity for large-scale production, their lower immunogenicity, and their ability to transfer large DNA plasmids and RNAs [7,9,13]. Meanwhile, their low transfection efficiency has been suggested as the main problem of these systems [14,15].

Among various nanocarriers, cyclodextrin (CD) has been identified as a promising and efficient platform for pharmaceutical drug delivery, specifically oligonucleotide-based therapy [16,17,18]. Cyclodextrins (CD), the monomer unit, are cyclic oligosaccharides containing glucopyranoside monomeric units linked via α-(1, 4)-glycosidic bonds with a central lipophilic cavity and outer hydrophilic surface [18]. Their application in the pharmaceutical and nutraceutical industries and similar fields has significantly increased in recent years [19,20,21]. In addition, natural CDs have been considered “generally recognized as safe” (GRAS) [22], and CD-based polymers are achieving promising applications [18,20]. They were first used as a plasmid DNA delivery system in 1999, and they were subsequently developed as siRNA carriers [23]. However, they could not directly interact with nucleic acids due to their weak interactions with these agents [24,25]. To improve this interaction, cationic CD derivatives and CD-based polymer structures bearing cationic sites have been used instead. The latter demonstrated valuable biocompatibility and protection of the therapeutic cargo by forming stable polyplexes with therapeutic nucleic acids [16,26].

In this regard, we synthesized novel cationic hyper-branched cyclodextrin-based polymers using choline chloride (CHO) to impart positive charges on the final product and carbonyldiimidazole (CDI) as the linking agent to be used as a delivery tool, in this case to deliver plasmid DNA encoding the Enhanced Green Fluorescent Protein (EGFP). We sought to compare the cytotoxicity and transfection efficiency of nanocomplexes with the standard lipofectamine in 2D (HT-29) and multicellular 3D spheroid cultures. Typically, conventional 2D cell culture models are used to understand the efficiency of gene delivery systems prior to investigating them in vivo. Despite the high transfection efficiency of genes through non-viral systems in monolayer two-dimensional (2D) cells, they have not been successful in providing therapeutic benefits in vivo due to the complex in vivo microenvironment, such as cell differentiation, proliferation, and gene expression, which cannot be fully replicated by 2D cell culture [27,28]. Therefore, there is need to introduce newly developed methods to fill this large gap between an in vitro 2D cell culture and in vivo animal (or human) models [29,30]. The supposed bridge to fill this gap between conventional 2D culture and animal models is three-dimensional (3D) spheroid cell culture, which has demonstrated more realistic cell-cell interactions, biochemical characteristics, and cellular parameters than 2D cell cultures. Such culture models are necessary for evaluating gene delivery systems before starting pre-clinical in vivo studies [31,32,33,34]. The differences in behavior between 3D spheroids and 2D cell culture have been demonstrated [35,36,37], and in some cases, drug candidates have demonstrated high activity in 2D culture while being inactive in 3D culture [28].

In the present study, we aimed to investigate the capacity of our newly synthesized cationic hyper-branched cyclodextrin-based polymers to be used as a gene delivery tool, in this case for the delivery of plasmid DNA encoding the EGFP, in 2D (HT-29) and multicellular 3D spheroid cell cultures in comparison with lipofectamine. Keeping the abovementioned studies in mind, the objectives were:

➢ To study the ability of positively charged NPs to retard the pDNA-eGFP at proper N/P ratios;
➢ To achieve enhanced transfection efficiency and cellular uptake of the pDNA-eGFP in complex with positively charged PPoly and to compare it with lipofectamine in 2D and 3D cell cultures;
➢ To understand the safety of NPs in both 2D and 3D cell cultures;
➢ To understand the differences among 2D and 3D spheroid cells in terms of the conditions for gene delivery.

## 2. Material and Methods

### 2.1. Material and Reagents

All chemical reagents were supplied from Sigma-Aldrich without additional purification. Human colorectal carcinoma cells HT-29 (NCBI Code: C466) and fibroblast were provided from the National Cell Bank of Iran (Pasteur Institute, Tehran, Iran). β-cyclodextrins (βCD) were provided by Roquette Freres (Lestrem, France), while choline chloride (CHO), carbonyldiimidazole (CDI), and dimethyl sulfoxide (DMSO) were purchased from Sigma-Aldrich (Darmstadt, Germany). Lysozyme from chicken egg white (62970), albumin from porcine serum (A1830), and bovine serum albumin (A3912) were purchased from Sigma-Aldrich (Darmstadt, Germany) and stored in a refrigerator. Furthermore, DreamFect ™ (DF40500) was provided from OZ Biosciences (Marseille cedex 09, France). βCD and choline chloride were dried in an oven at 75 °C up to constant weight before use. Coomassie brilliant blue (R-250) was obtained from Bio-Rad.

### 2.2. Polymer Synthesis

The synthesis of the polymer was carried out by dissolving, at room temperature, 1.00 g (8.81 × 10^−4^ mol) of anhydrous βCD in 7.5 mL of DMSO. After complete solubilization, 1.11 g (7.93 × 10^−3^ mol) of choline chloride was added followed by 1.14 g (7.05 × 10^−3^ mol) of carbonyl diimidazole. After a transparent liquid was obtained, the solution was kept stirring at 90 °C for 120 min; an increase in the viscosity was observed during that time, which demonstrated that the polymerization reaction was occurring. Afterwards, the product was precipitated and washed with acetone and then recovered by vacuum filtration. Next, the dry product was solubilized in distilled water and purified using an ultrafiltration technique (cutoff of 5k Da) to separate the polymer from unreacted reactants, byproducts, and solvent residues. The product was then recovered from the ultrafiltration cell and subsequently freeze dried, which resulted in a white powder as the product. Considering the weight of the final product with respect to the theoretical weight (equal to the sum of βCD, CHO, and CDI), the mass balance was approximately 85%.

#### 2.2.1. Elemental Analysis Characterization

The samples’ chemical composition was studied using a Thermo Fisher FlashEA 1112 Series elemental analyzer (Waltham, MA, USA).

#### 2.2.2. Size Exclusion Chromatography

Size exclusion chromatography analyses were performed using a PSS NOVEMA Max analytical linear S (Mainz, Germany) column installed on a Perkin-Elmer Flexar chromatograph (Akron, OH, USA). The mobile phase was a 0.1 M sodium chloride water solution + 0.3% formic acid: acetonitrile (80:20) at a flow rate of 1.0 mL/min. Refractive index was used as detector, while the duration of a single run was set at 15 min in isocratic conditions. A calibration curve was constructed before sample analysis using dextran standards with specific average molecular weights ranging from 6 kDa to 75 kDa.

### 2.3. Plasmid Encoding eGFP Preparation

The pmax plasmid (pmaxGFP) encoding enhanced green fluorescent protein was kindly gifted by Dr.Heidari, Medical Biotechnology department (TUOMS), Iran. The plasmid pmaxGFP was transformed in *E. coli* DH5α strain cultures and grown overnight in 2 L shake-flasks containing 250 mL of LB (Luria-Bertani) medium and antibiotics (30 mg/mL of Gentamicin). The purified plasmids were then obtained using a GenElutet Plasmid Miniprep EndoFree plasmid purification Kit (Sigma-Aldrich, Milan, Italy). The purified plasmid was diluted using tris-EDTA (TE) buffer solution and stored at −20 °C. The purity and concentration of the plasmid were determined by NanoDrop spectrophotometer (ThermoFisher, Waltham, MA, USA) absorbance at 260/280 nm.

### 2.4. Polymer–DNA Complex Formation and Gel Retardation Assay

The complex between cationic CD polymers and pmaxGFP were made before transfection experiments. Different N/P ratios (average number of nitrogen atoms on the cyclodextrin core/number of phosphate groups of pDNA) of polymer–DNA complexes were formed (0.5:1, 1:1, 6:1, 12:1, and 25:1) by mixing 10 µL of plasmid DNA solution (containing 1 µg of pmaxGFP pDNA in filtered distilled water) and 10 µL of polymer solution (containing varying amounts of polymer in filtered distilled water). To form the complexes, they were mixed and then vortexed for 10 s and incubated at room temperature for 30 min. Finally, obtained solutions were mixed with loading buffer and loaded onto a 1% agarose gel for gel electrophoresis, which was conducted in 1× TAE buffer (40 mmol/L Tris acetate and 1 mmol/L EDTA) at 90 V for 45 min. DNA bands were visualized by a UV trans-illuminator (Tamilnadu, India).

### 2.5. Complex Characterization

#### 2.5.1. Complex Size and Zeta Potential Measurement

Particle size and zeta potential measurement was performed on a 90 plus particle sizer (Malvern Instruments, Malvern, UK) (Zetasizer Ultra-Pro ZS Xplorer software update v3.20, Medium: ultrapure water, Dispersant RI: 1.330, Viscosity (cP): 0.8872). DNA–polymer complexes with different N/P ratios (0.5:1, 1:1, 6:1, 12:1, and 25:1) were formed in 0.5 mL PBS (Eppendorf tubes) by adding 10 µL of plasmid DNA solution (containing 1 µg of pmaxGFP pDNA in filtered distilled water) and 10 µL of polymer solution (containing varying amounts of polymer in filtered distilled water).They were then left for 30 min at room temperature to form the complexes and were then diluted to 1 mL with filtered distilled water. The particle size was measured at 25 °C in a glass cuvette.

#### 2.5.2. Scanning Electron Microscopy (SEM) Studies

SEM was used to visualize the polymer–DNA complexes and to obtain their optical and structural properties. The complexes were observed using SEM, and the gold-coated complexes were observed using a Cambridge Stereoscan 260 SEM (Cambridge, UK) at 20 kV.

#### 2.5.3. Fourier Transformed Infrared Study

Polymer alone, polymer–DNA complex, and free DNA were analyzed via Fourier transform infrared (FTIR) spectroscopic studies using a Bruker, Tensor 27 (Alexandria, VA, USA) instrument in the region of 400–4000 cm^−1^. This was conducted to understand the existence of interactions between polymer–DNA complexes. Samples were mixed with KBr and compressed under pressure to form discs.

#### 2.5.4. Determination of pDNA Encapsulation Efficiency

To check the encapsulation efficiency of the polymer for pDNA, an ultracentrifugation method was employed to determine the difference between the total amount of pDNA added in the polymer preparation buffer and the amount of free pDNA remaining in the aqueous suspension by NanoDrop spectrophotometer (ThermoFisher, Waltham, MA, USA) at 260 nm. The concentration of pDNA was determined using 1 (OD260 nm) = 50 μg of DNA [38].The pDNA encapsulation efficiency (EE) of the process was calculated from equations as indicated below:

(1) $$EE\%=\frac{\left[\left(a-b\right)\times \frac{d}{c}-\left(a-b\right)\right]}{a}\times 100$$

where *a* is the total amount of pDNA, *b* is the amount of free pDNA, *c* is the nanoparticle weight, and *d* is the total amount of polymer.

### 2.6. Cell Culture Protocol for 2D Cell Culture

The HT-29 (Passage Number: 3) cell line was obtained from Pasture Institute National Cell Bank of Tehran, Iran. The cells were cultivated in RPMI 1640 supplemented with 10% FBS (FBS, Gibco, Billings, MT, USA) and 1% Penicillin/Streptomycin solution (Biochrom GbmH, Berlin, Germany). The flask was incubated in a humidified incubator at 37 °C containing 5% CO_2_. Media was exchanged daily, and for each passage, the cells were detached and harvested after trypsinization at 80~90% confluence.

#### Cell Culture Protocol for 3D Spheroid

The multicellular 3D cancer cell spheroids were produced using the conventional hanging drop method [39]. Briefly, 1.5 × 10^4^ HT-29, 7.5 × 10^3^ HUVEC, and 7.5 × 10^3^ fibroblast cells were re-suspended in 20 μL of culture medium containing 1% FBS and placed at the inner surface of culture plate lids. The lid and drop containing cells were inverted over the culture plates. To prevent the evaporation of hanging drops, the plate was filled with phosphate-buffered saline. The plates were kept at 37 °C under a humidified condition with 5% CO_2_. After four days, when thick and compact micro aggregates with dark edges were generated, the spheroids were transferred into each well of 96-well plates containing 200 μL of culture medium with 1–2% FBS.

### 2.7. Cells Cytotoxicity Assay

#### 2.7.1. MTT (3-(4, 5-Dimethylthiazol-2-yl) 2, 5-diphenyl tetrazolium bromide) Assay

The MTT assay was used to determine in vitro cell viability according to a previous report with slight modifications [40]. HT-29 cells were seeded in 96-well plates and incubated until cells were 80% confluent, after which they were exposed to different N/P ratios of polymer–DNA complexes (0.5:1, 1:1, 6:1, 12:1, and 25:1) by mixing 10 µL of plasmid DNA solution (containing 1 µg of pmaxGFP DNA in filtered distilled water), 10 µL of polymer solution (containing varying amounts of polymer in filtered distilled water), and free plasmid DNA for 24 h.

#### 2.7.2. Lactate Dehydrogenase (LDH) Assay in 2D and 3D Cell Cultures

Cell membrane integrity was evaluated using the Plus Cytotoxicity Detection Kit method according to the manufacturer’s protocol with some modifications. Briefly, an HT-29 monolayer was seeded in a 96-well plate and incubated until cells were 80% confluent, and one multilayer 3D spheroid cell was seeded in each well. Cells were thereafter exposed to different N/P ratios (12:1, 25:1, 50:1, and 100:1) containing the same amount of free PPoly of plasmid for 24 h. The release of LDH to the supernatant culture medium was assessed to determine the occurrence of cell membrane injury. The micro-plate ELISA reader (Biotek, Santa Clara, CA, USA, Gen5™ software), at absorbance of 492 nm, was used to determine the concentrations of LDH.

### 2.8. Transfection Assessment in 2D and 3D Cell Cultures

#### 2.8.1. Measuring the DNA-Polymer Transfection Using Fluorescence Microscopy in 2D and 3D Cell Cultures

Trypsinized cells were seeded in a 6-well plate with a density of 1 × 10^6^ cells per well (2D cell culture model) and 3 × 10^4^ cells per 20 µL drop in 96-well plates (3D cell culture model) in RPMI medium supplemented with 10% FBS. After 24 h, the culture media was replaced with serum-supplemented media containing 1 and 2 μg of free pDNA-eGFP and complexes with PPoly at an N/P ratio of 25:1 for 2D and 3D cells, respectively. The cells were incubated with the complexes and naked DNA for 2, 4, 6, and 24 h in the 2D cell culture model, and the 3D cells were incubated for 6 and 24 h. Cells were then washed twice in PBS. For fluorescence microscopy observation (Cytation 5 Cell Imaging Multi-Mode Reader), cells were fixed by 4% paraformaldehyde followed by nuclear staining with 4′, 6-diamidino-2-phenylindole (DAPI) at room temperature for 30 min. Additionally, the DreamFect™ Transfection Reagent (Lipofectamine) was used at ratio 3:1 (DreamFect/pEGFP) according to the manufacturer’s protocol as a positive control for 24 h in both 2D and 3D cell culture.

#### 2.8.2. Measuring the DNA-Polymer Uptake Using Flow Cytometry Analysis

For this analysis, the monolayer 2D and multilayer spheroids cells were seeded in 12-well plates and incubated for 24 h. After 24 h, the culture media was replaced with serum-supplemented media containing 1 and 2 μg of free pDNA-eGFP and complexes with PPoly at an N/P ratio of 25:1 for 2D and 3D cells, respectively. The cells were incubated with the complexes and naked DNA for 2, 4, 6, and 24 h in the 2D cell culture model, and the 3D cells were incubated for 6 and 24 h. The 2D cells and spheroids were then washed twice with PBS before being disaggregated by incubating in a trypsin–EDTA solution. The cells were analyzed by the BD FACSCalibur™ Flow Cytometer system (Becton, Dickinson and Company, Franklin Lakes, NJ, USA) to calculate the fluoresce intensity of each group and were compared with the non-treated control cells. The raw data were analyzed using FlowJo software (ver. 7.6.1). Additionally, the DreamFect^TM^ transfection reagent (Lipofectamine) was used as a positive control for 24 h in both 2D and 3D cell culture at a ratio of 3:1 (Lipofectamine/pEGFP).

### 2.9. Histopathology Method for Evaluation DNA-Polymer Penetration in 3D Spheroid Cells

Prior to spheroid cell fixation, the cells were incubated with 2 μg of pDNA-eGFP in complexes with PPoly at an N/P ratio of 25:1 for 48 h. The cells were then washed twice with PBS and were subsequently stained with 4′, 6-diamidino-2-phenylindole (DAPI) at room temperature for 30 min. The following procedure was used to prepare the spheroid samples. Multilayer cancerous spheroid suspensions in media were collected from plates, placed into a micro-centrifuge tube, and spun at 1000 rpm for 2 min to form a pellet. After discarding the supernatant, phosphate-buffered saline (PBS) was used to wash the precipitated pellet, and centrifugation was conducted at 1000 rpm for 2 min to reform the pellet. The pellet was fixed in 10% neutral buffered formalin (NBF) for 10 min, followed by removal of fixative and replacement with PBS, and was stored at 4 °C until the time of sample preparation for processing and paraffin embedding. At the time of processing and embedding, the spheroid samples inside the micro-centrifuge tubes with PBS were centrifuged at 1000 rpm for 5 min. The PBS was removed, and a 1% agarose solution was pipetted into the micro-centrifuge tube containing the pellet, gently dispersed throughout the agar, and allowed to solidify for at least 1 h at room temperature. The intact spheroid pellets in agarose were then taken from the micro-centrifuge tubes. The suspended pellets were sliced longitudinally and placed into a tissue cassette with the cut side down. The pellets were processed on a tissue processor and infiltrated with 70% and 80% ethanol for 10 min each followed by 95% ethanol for 15 min and three changes of 100% ethanol for 10 min each. Next, the pellets were processed in three changes of xylene (10 min each), submerged in three baths of paraffin (15 min in the first bath and 25 min in each of the remaining two baths), and then placed (embedded) in a mold with paraffin. Sections of 5 mm diameter were cut and mounted onto hydrophilic glass slides. Finally, the hydrophilic glass slides were used to understand the penetration of pDNA-eGFP in complexes with cationic CD polymer in 3D spheroid cells by fluorescence microscopy observation.

### 2.10. Hemocompability

Hemolysis activity was tested based on the procedure of a previous study [41]. Male Wistar rats were used to collect blood cells in tubes containing ethylene diamine tetra acetic acid (EDTA) as an anti-coagulated agent. Red blood cells (RBCs) were isolated from serum by centrifugation (1000 rpm, 5 min). After the supernatant was removed, the pellet was washed several times with phosphate-buffered saline (PBS) (pH = 7.4) to obtain a final concentration of 2% (*v/v*). Next, 1 mL of RBC suspension was combined with 1 mL of PPoly-free with a concentration range of 125, 250, 500, and 1000 μg/mL and the polyplex complex with a range of different N/P ratios (0:1, 1:1, 6:1, 12.5:1, and 25:1). The experiments were approved by Tabriz university of medical sciences, Pharmacy department, Iran Ethical Code: IR.TBZMED.AEC.1401.022.

### 2.11. Statistical Analysis

Statistical analysis was conducted using GraphPad Prism 8 (GraphPad Software, Inc., La Jolla, CA, USA). Data were analyzed using one-way ANOVA (analysis of variance). All the samples were analyzed in triplicates and were presented as mean ± standard deviation (SD) for n = 5 or 3. The level of significance was calculated by the *p*-value. Statistically, *p* values < 0.05 were considered significant.

## 3. Results and Discussion

### 3.1. Synthesis and Characterization of Soluble Cyclodextrin-Based Polymer

Several methods have been reported for the production of soluble cyclodextrin-based polymers. This includes (i) grafting of cyclodextrins on previously synthesized polymers, (ii) polycondensation of difunctionalized cyclodextrins monomers obtained by selective modification of the cyclodextrins’ hydroxyl functions, (iii) quenching of the cross-linking process, and (iv) inhibition of the gelation process in a sol-gel cross-linking reaction [42,43,44]. According to Flory’s theory, gelation occurs when the branching coefficient (α) is higher than a critical value (αc) defined by the following equation:

(2) $$\alpha_{C}=\frac{1}{f-1}$$

where *f* is the number of functional groups in the polyfunctional monomer.

**Figure 1:**
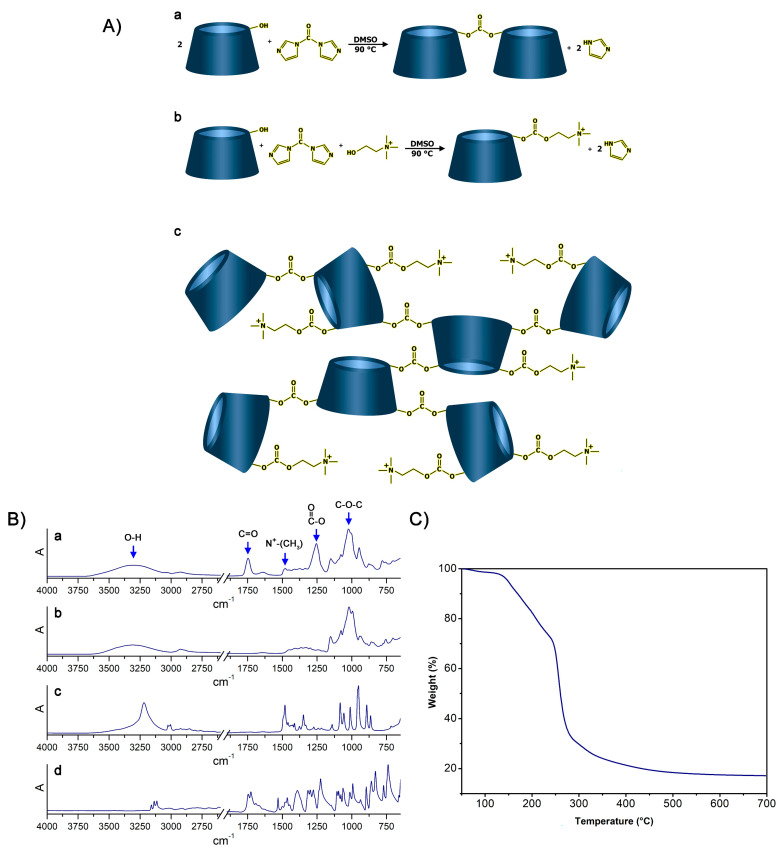
(**A**): (**a**) Reaction occurring between βCD and CDI, (**b**) reaction occurring between βCD, CDI, and CHO, (**c**) structure of the resulting hyper-branched polymer. (**B**): FTIR-ATR spectra of (**a**) PPoly, (**b**) βCD, (**c**) CHO, and (**d**) CDI. (**C**) TGA of PPoly.

In this regard, the possibility to synthesize cationic hyper-branched cyclodextrin-based polymers while avoiding the production of cross-linked structures was demonstrated. With the goal of obtaining a βCD-based polyelectrolyte, CHO was used to impart positive charges to the final product. The presence of the positive charge as a CHO pendant can increase the capacity of the material to bond DNA [45]. Moreover, CHO has been extensively reported for its non-toxicity, and, especially in recent years, it has been widely exploited for the preparation of the so-called NADES (natural deep eutectic solvents) [46,47,48]. As reported in Figure 1A, the presence of a hydroxyl function in its structure allowed CHO to display a similar reactivity to that displayed by βCD towards the linker, which was CDI in this case. CDI allowed the formation of carbonate bridges by linking (i) βCD units together or (ii) βCD to CHO molecules. As a result of the abovementioned reactivity paths, the formation of a cationic hyper-branched polymer structure was hypothesized. The formation of a polymer structure was first observed by the presence of a product after the purification step, which was performed using an ultrafiltration technique with a 5k Da cutoff membrane. The amount of purified polymer (PPoly) obtained after freeze-drying the retentate was expressed as mass balance and resulted in roughly 85 wt% with respect to the weight of the polymer before the ultrafiltration process. The molecular weight of PPoly was subsequently studied via size exclusion chromatography. As a result, a molecular weight of 22 kDa was observed. Furthermore, the FTIR-ATR spectra of PPoly (Figure 1B) clearly displayed a peak centered at 1747 cm^−1^ (C=O stretching) and a peak at 1255 cm^−1^ (C-O stretching), both of which were associated with the presence of carbonyl groups belonging to carbonate bridges [49,50], as hypothesized in Figure 1. Additionally, a peak centered at 1479 cm^−1^ (N^+^-CH_3_ bending) [51], associated with the presence of quaternary ammonium functions, was a further proof of the mechanism previously described. Subsequently, the presence of quaternary ammonium pendants was studied via elemental analysis since the presence of nitrogen atoms within the polymer structures can be related only to the presence of CHO pendants. As reported in Table 1, the presence of ammonium pendants was supported by elemental analysis results. Furthermore, by assuming that all the CHO introduced during the synthesis ended up bound within the polymer network and comparing it with the nitrogen content obtained via elemental analysis, it was possible to calculate the actual fraction of CHO present in the final product compared with the theoretical 100% value. The amount of CHO bound to PPoly was roughly 80% of the amount introduced during the synthesis. This feature might be related to either non-reacted CHO or CHO bound to oligomer chains characterized by molecular weights lower than 5k Da, which thus would have been separated from the purified product during the ultrafiltration step. Additionally, the thermal stability of PPoly was studied via TGA. As reported in Figure 1, PPoly displayed a Tonset at approximately 150 °C followed by a two-step degradation taking place first between 150 °C and 250 °C and then from 250 °C to 450 °C. This gave a final carbon residue corresponding to approximately the 20% of the initial weight (Figure 1C).

### 3.2. Preparation and Characterization of PPoly-DNA Complexes

**Figure 2:**
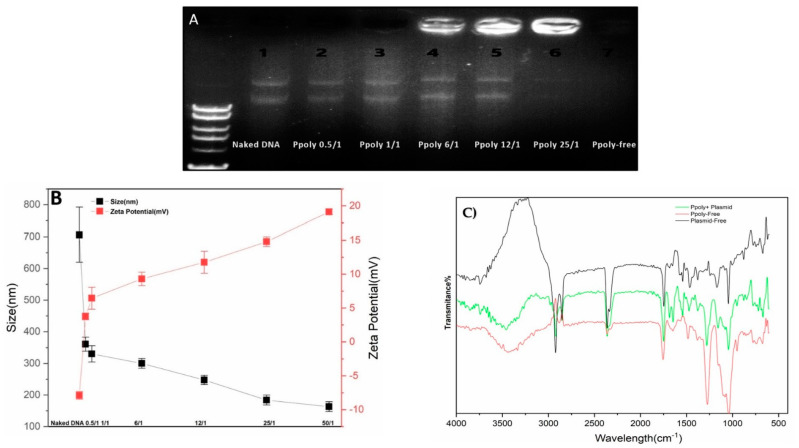
Characterization of PPoly–DNA nanocomplexes. (**A**) Agarose gel electrophoresis of PPoly–DNA nanocomplexes; (**B**) size of PPoly–DNA nanocomplexes at different N/P ratios and zeta potential of DLS; (**C**) the FTIR spectra of free PPoly, free DNA, and PPoly–DNA nanocomplexes. The results are expressed as means ± SD.

DNA condensation capability is a prerequisite property for a vector to act as an efficient gene delivery agent [52]. Thus, electrophoretic mobility shift assays (EMSA), particle size and zeta potential measurements, and SEM imaging were conducted to confirm the DNA condensing ability of the prepared polymer nanoparticles. The PPoly and DNA nanocomplex were made through electrostatic interactions of negatively charged pDNA with positively charged PPoly by CHO [45,53]. As shown in Figure 2A, the plasmid DNA was not completely condensed by PPoly at lower N/P ratios, whereas the migration of DNA was completely retarded when the N/P ratio was 25:1, which indicated the complete complexation of all DNA by PPoly through ionic interactions [54]. The hydrodynamic size and surface charge of non-viral carriers are the most important factors to consider for drug and gene delivery. Therefore, the average particle size and zeta potential of the PPoly–DNA complexes were measured by dynamic light scattering (DLS). As can be seen in Figure 2B, by increasing the N/P ratios, the average particle size and zeta potential of PPoly–DNA complexes decreased and increased gradually, respectively. At low N/P ratios of 0.5:1 and 1:1, the particle sizes were higher at 360.5 ± 21.9 and 330 ± 25.4, respectively; a possible explanation for this phenomenon may be due to incomplete DNA complexation with PPoly at these ratios. The results indicated that when the polymer/pDNA weight ratio reached 25:1, the PPoly could effectively condense pDNA into a nanosized particle (<200 nm) with a positive ζ-potential (approximately + 15 mV). Notably, it has been reported that polymeric NPs with a diameter of 100–200 nm have the best properties for preventing phagocytic uptake [55]. Additionally, the particle size and morphology of the PPoly–DNA complex at that ratio was further characterized by SEM (Figure S1). The SEM image also showed that the particle sizes of PPoly–DNA complexes were around 37–58 nm and were spherical in shape, which was well correlated with the DLS data. In addition, the FTIR spectra for the N/P ratio of 25:1 confirmed the effective loading of DNA–polymer complexes inside the CD NP (Figure 2C). For example, the abundant peak at 3500 nm^−1^ (-O-H) is removed for the complexation. In addition, the PO_3_^−2^ peak of DNA (around 1250 nm^−1^) or base/in-plane vibration (around 1600 nm^−1^) are shifted [56]. Therefore, according to the obtained data, the formulation at the N/P ratio of 25:1 was selected for cellular transfection analysis.

### 3.3. Encapsulation Efficacy and Hemocompatibility

**Figure 3:**
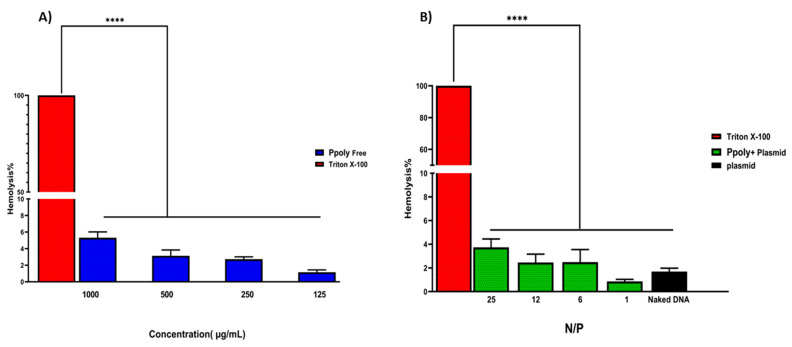
(**A**) Hemolysis percentages of RBCs at the concentration range of 125–1000 μg/mL of free PPoly. PBS and Triton X-100 were used as negative and positive control, respectively. (**B**) The Hemolysis percentages of PPoly/pDNA with different N/P ratios (1, 6, 12, and 25) and free pDNA. The data is provided as mean ± S.D. (n = 3). **** *p* ˂ 0.0001 (n = 3).

The encapsulation efficiency of plasmid DNA inside the PPoly at a ratio of 25:1 was about 85%, which might explain the pDNA complete condensation by the provided nanoparticle at that ratio. Another important assessment was the evaluation of the hemocompatibility of NP formulations, as this is a crucial parameter for their use in in vivo experiments [57,58]. The supporting hypothesis for the hemolysis activity of nanocarriers is that when blood contacts cationic macromolecules, the initial adsorption by plasma proteins leads to adhesion and activation of platelets, which in turn cause hemolysis, thrombosis, and embolization [59]. The free formulations showed hemolytic activity from about 1.2 to 5.5% at polymer concentrations of 125 to 1000 µg/mL (Figure 3), while these values were reduced to less than 0.8 to 4% when the nano-formulation was complexed with pDNA at ratios of 1:1 to 25:1. A potential explanation may be that electrostatic interactions between the polymer and pDNA lead to a decrease in the net positive charge of the polymer to reduce the interaction of the polymers with the negatively charged red blood cells, leading to a decrease in hemolysis [60]. Thus, the results of the presented experiment illustrated that our NPs are hemocompatible with rats’ blood.

### 3.4. Cytotoxicity Assay

#### 3.4.1. MTT Assay in 2D Cell Culture

**Figure 4:**
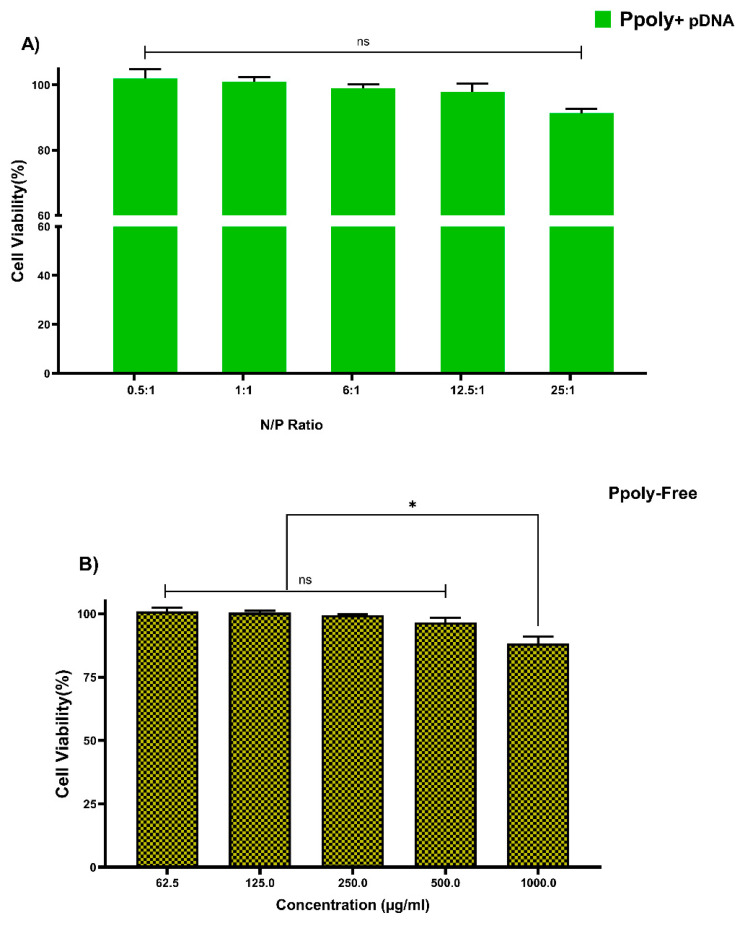
Measuring the viability of HT-29 cells using MTT assay in a 2D culture system after 24 h. (**A**) PPoly in complexes with pDNA at different N/P ratios. (**B**) free PPoly at concentrations 62.5 to 2000 µg/mL. One-way ANOVA with Tukey post hoc analysis was performed. * *p* < 0.05 and ns (not significant).

Cytotoxicity of free PPoly and complexes with pDNA, which is one of the most important concerns for the use of non-viral gene delivery vehicles, was evaluated in the HT-29 cell line using the MTT assay. The cells were treated with the same N/P ratios and concentrations used in the hemolysis tests. The different concentrations of free PPoly (62.5, 125, 250, and 500 μg/mL) did not show the significant cytotoxicity against HT-29 cancer cells, and we observed negligible cytotoxicity of βNS-CDI 1:4 against that cell line at 500 μg/mL [19]. However, free PPoly showed around 12% cytotoxicity at 1000 µg/mL (Figure 4A). On the other hand, the cells treated with polyplexes (pDNA/Ppoly) at low N/P ratios had a negligible (around 4%) cell toxicity, and the toxicity was around 10% at a ratio of 25:1 (Figure 4B). The results indicated that this nanoparticle, at the ratio selected for biological activity (25:1), is safe and with a negligible cytotoxicity, although they displayed a higher cytotoxicity (about 42 and 35% for free PPoly and complexes with pDNA, respectively) at a ratio of 50:1 and concentration of 2000 μg/mL. This elevated cell death rate was heightened by increasing the amount of total positive charge of the carrier, as the cytotoxicity of Tetraiodothyroacetic acid conjugated polyethylenimine (PEI) has been reported to be increased by raising the positive charge density on the surface of PEI [61]. This induced greater cytotoxicity, and this issue may merely be explained by greater interaction between the negatively charged components on the cell membrane and the high positive charge of the polymer, which leads to the disruption of plasma membrane and, consequently, cell death [59,61,62,63,64,65].

#### 3.4.2. LDH Assay in 2D and 3D Cell Cultures

**Figure 5:**
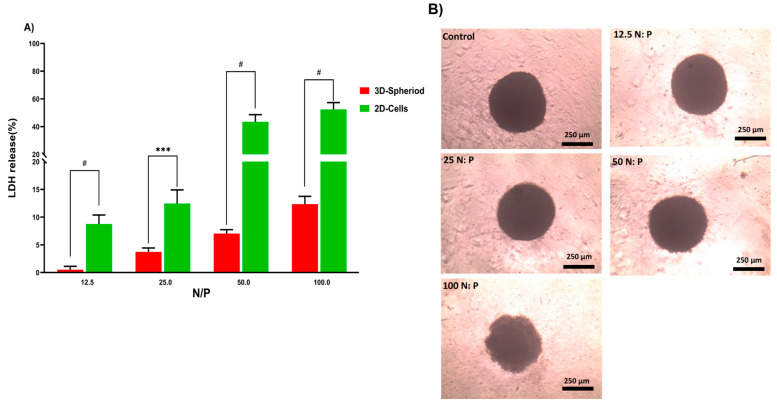
(**A**) Evaluation of LDH release from HT-29 2D and 3D spheroid cells after 24 h exposure to PPoly–pDNA complexes at 12.5, 25, 50, and 100 N/P ratios. (**B**) The incubation of spheroids with different ratios of PPoly–pDNA complexes contributing to the loss of spheroid integrity and structure. One-way ANOVA with Tukey post hoc analysis was conducted. *** *p* < 0.001 and # *p* < 0.0001.

In the next step, the lactate dehydrogenase (LDH) release by 2D and 3D cells treated with PPoly in complex with pDNA at different ratios was evaluated to understand the toxicity effects. LDH release is considered an indicator of plasma membrane damage because when the cell membranes are compromised or damaged in any way, LDH, a soluble yet stable enzyme found inside every living cell, is released into the surrounding extracellular space. This extracellular protein assay was performed on the supernatant of both 2D and spheroid cell cultures after 24 h exposure to the PPoly in complex with pDNA at different ratios. The data showed that increasing the NP concentration increased LDH release; the amount of LDH released in 2D cells increased from about 10 to 60% in complexes with N/P ratios ranging from 12.5:1 to 100:1 (Figure 5A). In addition, the LDH release in 3D cell culture was slightly increased from 1 to 12% with the ratio of 12.5:1 to 100:1, respectively. These data indicated that the LDH leakage in 3D cell culture was lower about five times at all ratios compared to the 2D cells (*p* < 0.0001). However, this large gap between the 2D and 3D spheroid cells in terms of their sensitivity to different ratios of complexes and the release of LDH content was expected due to the nature of the different cell phenotypes. For example, the cells in 2D culture are more exposed to the solution, while approximately all the cells in spheroid culture are enclosed by the outer layer of cells and would not make direct contact with the solution interface. Therefore, 2D cells are more sensitive to leakage of their cytosolic contents into the media [66,67]. Another study showed that spheroid cells were considerably less sensitive than 2D cells when the cells were treated with the chemo-therapeutic agent Taxotere [68]. Furthermore, the integrity of spheroid HT-29 cells was examined in the same ratios defined in the LDH assay to understand the effect of the complex on the cell’s density and integrity after 24 h exposure. The results showed that only at the last ratio did the spheres begin to disintegrate slightly and lose their central density compared with controls (Figure 5B). One explanation could be that exposure of HT-29 tumor cells to complexes in a specific ratio of 100:1 can lead to a weakening of structural consistency and help reduce cell-to-cell attachment and intercellular attachment, thereby increasing the LDH leakage of 3D spheres at this ratio more than others [69]. Given that various studies have shown that 3D and 2D cell cultures have different sensitivities to induced toxicity [70,71,72,73,74], 3D cell culture and tumoroid formation can mimic in vivo cell behavior to some extent but not completely.

### 3.5. Cellular Uptake and Transfection Analysis in 2D Culture

**Figure 6:**
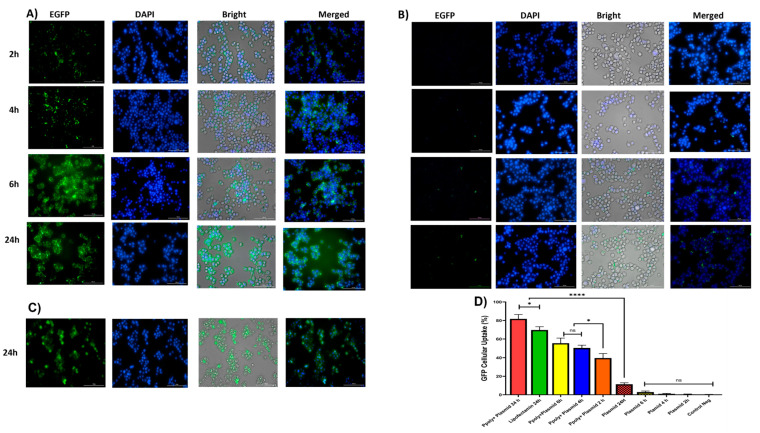
(**A**) Microscopic fluorescent images of 2D cells after 2, 4, 6, and 24 h incubation with PPoly–pEGFP nanocomplexes at an N/P ratio of 25:1. (**B**) Microscopic fluorescent images of 2D cells transfected with free pDNA after 2, 4, 6, and 24 h. (**C**) Microscopic fluorescent images of 2D cells transfected with lipofectamin/pEGFP at a ratio of 3:1 after 24 h. (**D**) Quantitative transfection efficacy (%) determined by the EGFP content as assessed by FACS analysis of treated 2D cells with PPoly–pEGFP nanocomplexes at an N/P ratio of 25:1 compared with free pDNA after 2, 4, 6, and 24 h and lipofectamine after 24 h incubation. Representative measurements of three independent experiments are reported. * *p* < 0.05 and **** *p* < 0.001.

Quantitative and qualitative analyses were performed to analyze intracellular transfection and localization in vitro in 2D monolayer cells. Two-dimensional cells were incubated with complexes containing 1 μg/mL of pDNA-eGFP at an N/P ratio of 25:1 for 2, 4, 6, and 24 h. As shown in Figure 6A,B, cells transfected with PPoly formulation loaded pDNA showed stronger green fluorescence related to EGFP protein expression compared with free pDNA and lipofectamibe as a positive control (Figure 6C), which showed moderate EGFP protein expression. It is worth noting that the strong green fluorescence inside the cells is related to the cells’ ability to absorb our NPs. The quantitative uptake of complexes containing 1 μg/mL of pDNA-eGFP at an N/P ratio of 25:1 was assessed by flow cytometry (FACS) analysis (Figure 6 and Figure S2) after incubation for the same durations used in the fluorescent microscopy experiments, and the efficacy of uptake was assessed. While free pDNA showed low cell uptake of less than 3% for 2, 4, and 6 h and about 12% after 24 h in HT-29, PPoly obtained about 40, 50, 55, and 65% at 2, 4, 6, and 24 h, respectively. In addition, lipofectamine showed about 70% efficacy of uptake after 24 h incubation, but this percentage was less than PPoly at the same time (Figure 6D and Figure S1). These data showed that PPoly loaded with DNA plasmid had good transfection efficiency, which increased over time by prolonging the plasmid release of nanoparticle compared to free pDNA and lipofectamine (Figure 6D).

### 3.6. Cellular Uptake and Transfection Analysis in 3D Spheroid Culture

**Figure 7:**
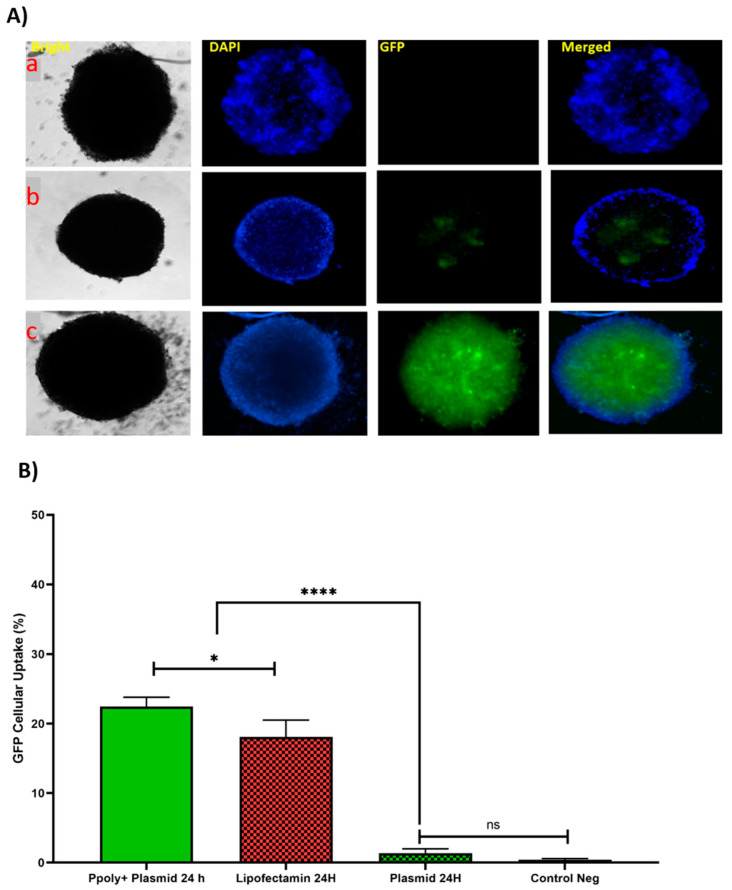
(**A**) Microscopic fluorescent images of 3D spheroids with PPoly/pEGFP nanocomplexes transfection at an N/P ratio of 25:1 (**c**) in comparison with free pDNA (**a**) and lipofectamine (**b**) after 24 h incubation. (**B**) Quantitative transfection efficacy (%) determined by the EGFP content as assessed by FACS analysis of 3D cells treated with PPoly/pEGFP nanocomplexes at an N/P ratio of 25:1 in comparison with free pDNA and lipofectamine after 24 h incubation. Representative measurements of three independent experiments are reported. The *p*-values are indicated as * <0.05, **** <0.0001, and ns (not significant).

In the next step, the ability of our NPs to penetrate into a 3D spheroid structure was evaluated. Spheroids were prepared by mixing HT-29, HUVEC, and fibroblast cells as a model for the study of uptake and transfection of our pDNA-loaded PPoly in comparison with free pDNA and lipofectamine. The tumorous spheroid cells were exposed to complexes containing 1 µg/mL of pDNA-eGFP at an N/P ratio of 25:1 for 24 h, and the free pDNA with the same concentration of lipofectamine was used at ratio 3:1 (DreamFect/pEGFP) at the same time. Qualitative observations using fluorescent microscopy revealed that our NPs showed a much greater capacity to penetrate the spheroid structure and induce transfection compared with free pDNA and lipofectamine after 24 h of incubation (Figure 7A).

The quantitative uptake of complexes containing 1 μg/mL of pDNA-eGFP at an N/P ratio of 25:1 was determined by flow cytometry (FACS) analysis (Figure 7B and Figure S3) after incubation for the same durations used in the fluorescent microscopy experiments, and the efficacy of uptake was found to be about 23% and 17% at 24 h for pDNA loaded on PPoly and lipofectamine, respectively. Although free pDNA did not show cell uptake after 24 h of incubation with the spheroid cells, the extracellular matrix (ECM) and high cell density multicellular barriers in the deep regions of the 3D spheroids inhibited the gene delivery agents’ penetration inside the spheroids, resulting in lower EGFP expression in 3D spheroid cells transfected by PPoly-pDNA complexes at a given time. Other studies on gene delivery in 3D spheroids showed a similar low transfection efficiency [75,76,77,78]. These cells are commonly known to exhibit extensive cell contact with simultaneous increases in interstitial pressure and significant resistance to chemotherapy and radiotherapy. This may also be true for the non-viral gene delivery systems. It is a well-known problem that transfection efficiency and cytotoxicity of non-viral gene delivery are closely related, suggesting that higher transfection efficiencies may be related to higher cytotoxicity. In our study, we observed this phenomenon and showed that it is true for transfection in both two-dimensional cells and three-dimensional spheroids. Our NPs demonstrated high transfection efficiency when examined in conventional 2D monolayer cells (HT-29), while they showed low transfection efficiency in 3D spheroids. The possible explanation would be that only parts of the peripheral cells (the outermost cell layers) in 3D spheroids were transfected by PPoly–pDNA complexes (N/P ratio of 25:1). These results are consistent with the results of other studies on the in vitro 3D spheroid model for gene delivery [78,79,80], and it also justifies clinical results that have shown low non-viral gene delivery efficiency in solid tumors or three-dimensional architectural tissues in vivo [79].

### 3.7. Evaluation the Penetration Capacity of PPoly–pDNA Complexes inside the 3D Spheroid Cells on Paraffin-Embedded Cell Block

**Figure 8:**
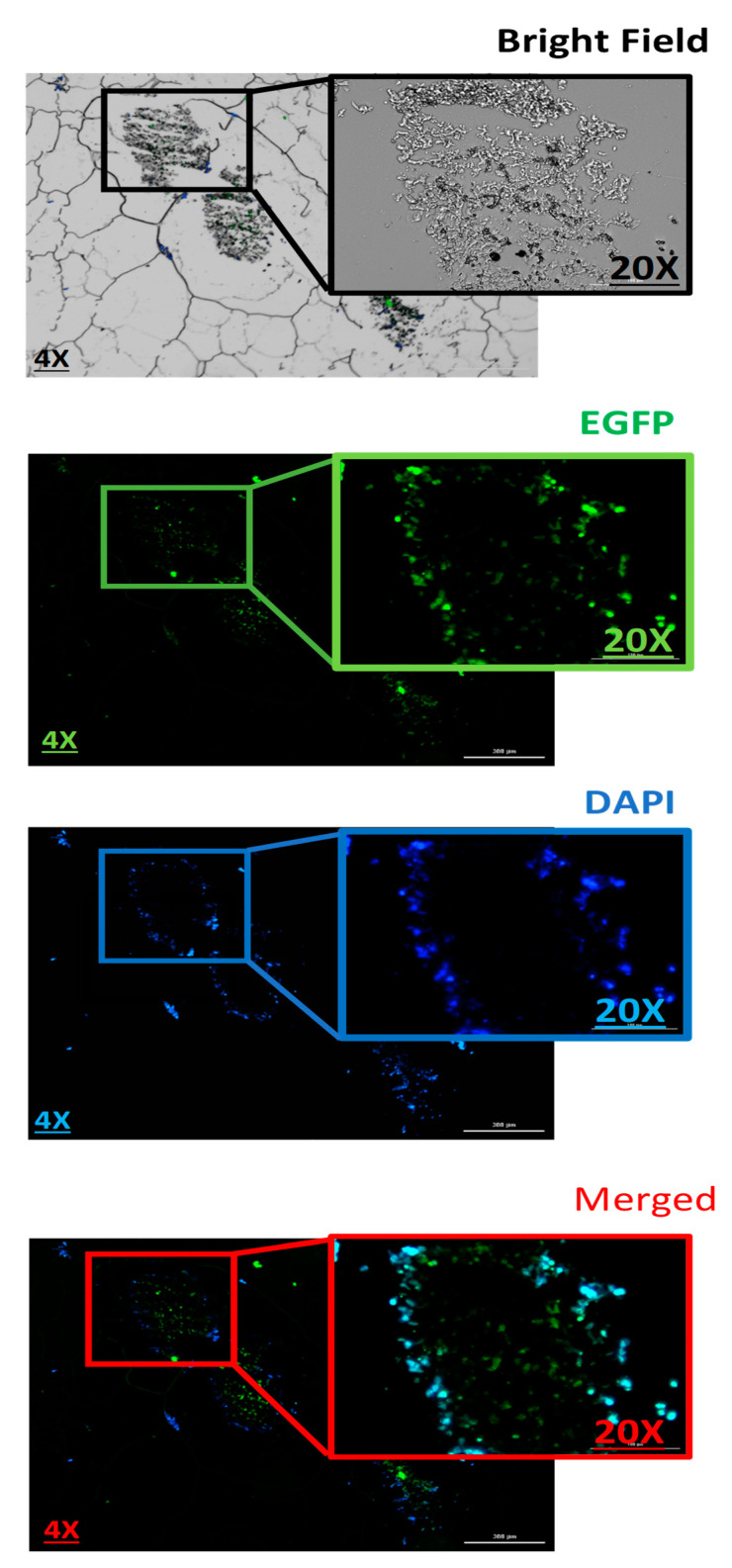
Evaluation the 3D spheroid cells on paraffin-embedded cell block to confirm the spatial expression of EGFP inside the spheroids. Scale bars: 4× and 20×. DNA refers to the pmaxGFP plasmid.

Next, histopathology was used to evaluate the pDNA-polymer penetration in 3D spheroid cells by incubating the cells with 2 μg of pDNA-eGFP in complexes with PPoly at an N/P ratio of 25:1 for 48 h. As shown in Figure 8, the EGFP fluorescent was observed, which indicated the capacity of our NPs to penetrate into the inner layers of the spheroid cells. Moreover, while DAPI dyes remained in outlier cells due to their inability to penetrate inside the spheroid. This result supported our previous experiments and demonstrated the ability of our NPs to carry the pDNA inside the cells in 2D and 3D cultures.

## 4. Conclusions

We synthetized new cationic hyper-branched cyclodextrin-based polymers, encapsulating plasmid DNA encoding EGFP proteins as a model nucleic acid to demonstrate the uptake and transfection efficiency of nanoparticles and their application in two-dimensional and three-dimensional cell cultures. The gel retardation assay illustrated that PPoly-loaded pDNA was effectively retarded at an N/P ratio of 25:1. Furthermore, PPoly-loaded pDNA was taken up more than 80% and 20% by cells in 2D and 3D cell culture models, respectively. Meanwhile, pDNA cellular uptake at both 2D and 3D cells was greater in complexes with PPoly compared with lipofectamine after 24 h. Interestingly, the results of the histopathology confirmed the ability of our PPoly to penetrate into and allow the transfection of a 3D spheroid. Furthermore, cytotoxicity (MTT and LDH) assay outcomes showed that our NPs were non-cytotoxic at a defined N/P ratio (25:1), specifically in 3D spheroid culture. Hemolysis results revealed that the complexes of PPoly and pDNA were hemocompatible when exposed to the red blood cells of rats, as they produced a lysis rate less than 4%. Although the quantification data of cellular uptake illustrated that EGFP expression in 3D spheroid cells transfected by PPoly–pDNA complexes at a determined ratio was lower than in 2D cells, this phenomenon may be due to the extracellular matrix (ECM) and high cell density multicellular barriers in the deep regions of the 3D spheroids, which inhibit the gene delivery agents’ penetration inside the spheroid cells. Taken together, thanks to the properties of our NPs (e.g., biocompatibility, hemocompatibility, and considerable cellular uptake to transfect the pDNA inside the cells), they might be considered as an efficient and promising platform for gene delivery in 2D and 3D mediums.

## Figures and Tables

**Table 1:** Elemental analysis of PPoly.

Element	wt%
N	3.28 ± 0.32
C	39.26 ± 0.55
H	6.56 ± 0.16
O	50.9 ± 0.12

## Data Availability

Not applicable.

## Supplementary Materials

The following supporting information can be downloaded at: https://www.mdpi.com/article/10.3390/pharmaceutics14122690/s1, Figure S1: SEM image of polymer/pDNA at N/P ration of 25:1. The image has confirmed the ability of the prepared polymer nanoparticles to condensing the pDNA in a spherical shape with the particle sizes around 37–58 nm; Figure S2: Quantitative transfection efficacy determined by the EGFP content as assessed by FACS analysis of treated 2D cells with CD/pEGFP nanocomplexes 2(B), 4(C), 6(D) and 24(E) hours at N/P ratio 25:1in compared to free pDNA after 2(F), 4(G), 6(H) and 24(I) hours incubation and control (A); Figure S3: Quantitative transfection efficacy determined by the EGFP content as assessed by FACS analysis of treated 3D cells with CD/pEGFP nanocomplexes at N/P ratio 25:1 in 6(B) and 24(C) hours compared to free pDNA after 6(D) and 24(E) h incubation and control(A).

## Abbreviations

PPoly: Purified polymer, CDI: 1, 1′-carbonyldiimidazole, CHO: Choline chloride, DMSO: Dimethyl sulfoxide, DLS: Dynamic light scattering, ATR: Attenuated total reflectance, SEM: Scanning electron microscopy, TGA: Thermogravimetric analysis, UV: Ultraviolet-visible spectroscopy, FTIR: Fourier-transform infrared, EMSA: Electrophoretic mobility shift assays, ELISA: Enzyme-linked immunosorbent, DAPI: 4′, 6-diamidino-2-phenylindole, EGFP: Enhanced Green Fluorescent Protein, EE: Encapsulation Efficiency, FBS: Fetal bovine serum, FACS: Fluorescence-activated Cell Sorting, LDH: Lactate dehydrogenase, MTT: 3-(4, 5-dimethylthiazol-2-yl) 2, 5-diphenyl tetrazolium bromide, pDNA: Plasmid DNA, PBS: phosphate-buffered saline, TAE: Tris-acetate-EDTA, 2D: Two dimensional, 3D: Three dimensional.
