# Supplementary material for: Hyper-Branched Cationic Cyclodextrin Polymers for Improving Plasmid Transfection in 2D and 3D Spheroid Cells
Source: Pharmaceutics. 2022 Dec 1;14(12):2690. doi: 10.3390/pharmaceutics14122690 (PMC9785855; doi:10.3390/pharmaceutics14122690)
Supplement: Supplementary file 1 [file pharmaceutics-14-02690-s001.zip › pharmaceutics-2030839-supplementary.pdf]

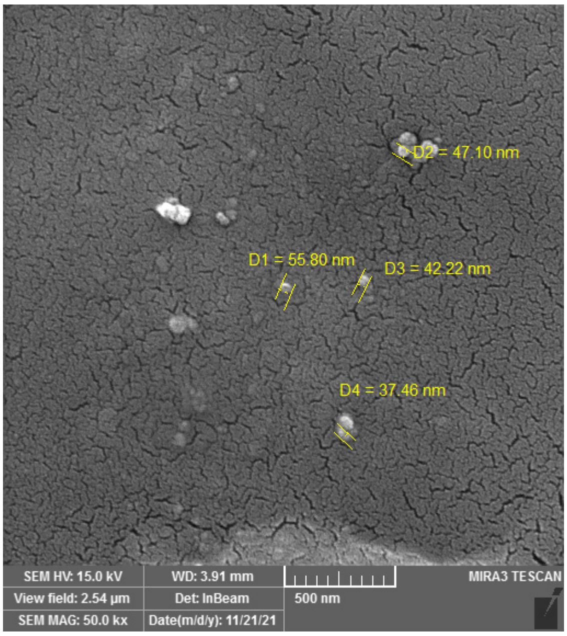

Figure S1. SEM image of polymer/pDNA at N/P ration of 25:1.

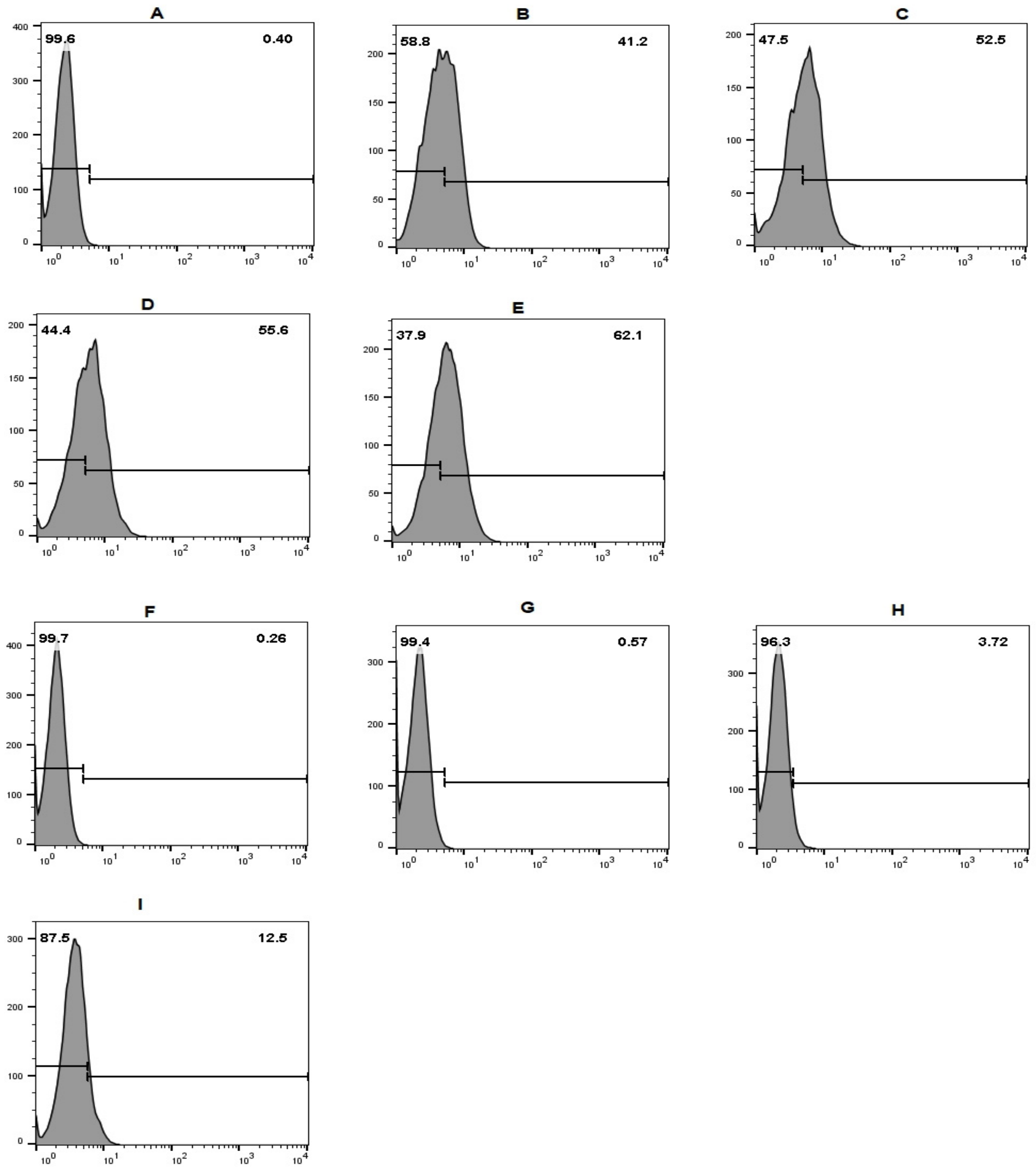

Figure S2. Quantitative transfection efficacy determined by the EGFP content as assessed by FACS analysis of treated 2D cells with CD/pEGFP nanocomplexes 2(B), 4(C), 6(D) and 24(E) hours at N/P ratio 25:1in compared to free pDNA after 2(F), 4(G), 6(H) and 24(I) hours incubation and control (A).

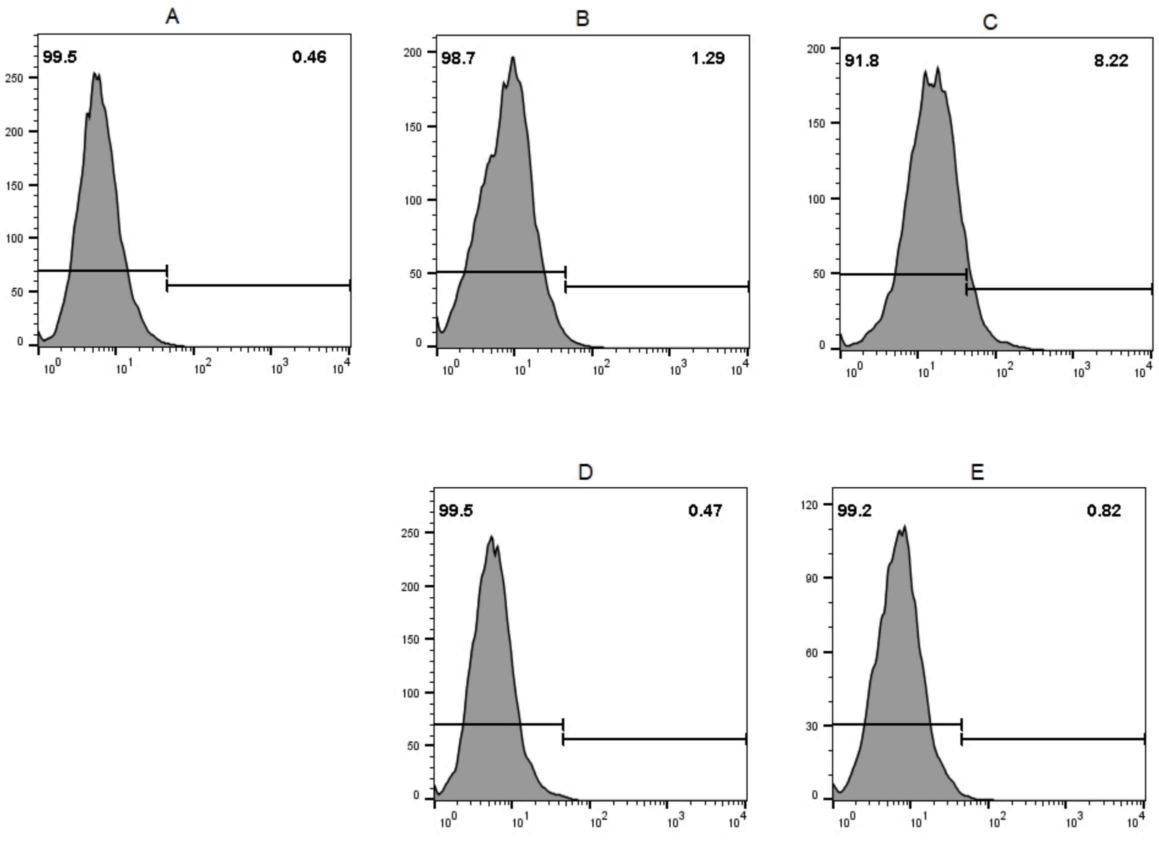

Figure S3. Quantitative transfection efficacy determined by the EGFP content as assessed by FACS analysis of treated 3D cells with CD/pEGFP nanocomplexes at N/P ratio 25:1 in 6(B) and 24(C) hours compared to free pDNA after 6(D) and 24(E) h incubation and control(A).
